# Tailored educational intervention for primary care to improve the management of dementia: the EVIDEM-ED cluster randomized controlled trial

**DOI:** 10.1186/1745-6215-14-397

**Published:** 2013-11-20

**Authors:** Jane Wilcock, Steve Iliffe, Mark Griffin, Priya Jain, Ingela Thuné-Boyle, Frances Lefford, David Rapp

**Affiliations:** 1 Research Department of Primary Care & Population Health, University College London, Royal Free Campus, Rowland Hill Street, London NW3 2PF, UK

**Keywords:** Dementia, Memory disorders, General practice, Primary care, Family medicine, Information management, RCT, UK

## Abstract

**Background:**

Early diagnosis of dementia is important because this allows those with dementia and their families to engage support and plan ahead. However, dementia remains underdetected and suboptimally managed in general practice. Our objective was to test the effect of a workplace-based tailored educational intervention developed for general practice on the clinical management of people with dementia.

**Methods:**

The tailored educational intervention was tested in an unblinded cluster randomized controlled trial with a pre/post-intervention design, with two arms: usual/normal care control versus educational intervention. The primary outcome measure was an increase in the proportion of patients with dementia who received at least two documented dementia-specific management reviews per year. Case identification was a secondary outcome measure.

**Results:**

23 practices in South-East England participated. A total of 1,072 patients with dementia (intervention: 512, control: 560) had information in their medical records showing the number of reviews within 12 months (or a proportion of) before intervention or randomization and within 12 months (or a proportion of) after. The mean total number of dementia management reviews after the educational intervention for people with dementia was 0.89 (SD 1.09; minimum 0; median 1; maximum 8) compared with 0.89 (SD 0.92; minimum 0; median 1; maximum 4) before intervention. In the control group prior to randomization the mean total number of dementia management reviews was 1.66 (SD 1.87; minimum 0; median 1; maximum 12) and in the period after randomization it was 1.56 (SD 1.79; minimum 0; median 1; maximum 11). Case detection rates were unaffected. The estimated incidence rate ratio for intervention versus control group was 1.03 (*P* = 0.927, 95% CI 0.57 to 1.86).

**Conclusions:**

The trial was timely, coinciding with financial incentives for dementia management in general practice (through the Quality Outcomes Framework); legal imperatives (in the form of the Mental Capacity Act 2005); policy pressure (The National Dementia Strategy 2009); and new resources (such as dementia advisors) that increased the salience of dementia for general practitioners. Despite this the intervention did not alter the documentation of clinical management of patients with dementia in volunteer practices, nor did it increase case identification.

**Trial registration:**

NCT00866099/Clinical Trials

## Background

Timely diagnosis can relieve the significant psychological distress that people with dementia and their close supporters experience. It allows them to engage with medical and psychosocial support, acquire or strengthen positive coping strategies, fulfill short-term goals and plan for the future, all of which can maintain and/or improve functioning and morale [1-4].

Efforts to improve the identification and diagnosis of dementia should be targeted at primary care, as this is the first point of contact for most individuals and their caregivers. However, dementia presents many challenges for primary care [5-7] and it remains underdetected and suboptimally managed [8]. General practitioners in England consistently report limited skills and confidence in diagnosis and management of dementia [9], and a minority see this as a task only for specialists [10]. An educational intervention to improve the skills of practitioners in the recognition of, and response to, dementia syndromes would be expected to be beneficial. However, in the absence of organizational change in health services and incentivisation of high quality care, evidence suggests that education alone is unlikely to change practice [11].

Since 2000, a natural experiment has occurred in the English National Health Service (NHS) via the introduction of policies and financial incentives for dementia diagnosis and management that have made a trial of an educational intervention in primary care to improve clinical practice particularly timely. Evidence-based policy imperatives, including guidelines from the National Institute for Health and Care Excellence (NICE) [8], are driving NHS management to prioritize dementia, especially earlier diagnosis and better clinical management in the community (the English Dementia Strategy [12]). Since 2006 changes in the reimbursement of primary care physicians through the Quality and Outcomes Framework (QOF) have incentivised the diagnosis and management of dementia syndrome [13], rewarding construction of a dementia disease registry and completion of an annual review of each patient with dementia (see Table 1). These policy and financial incentives for dementia diagnosis and management have created an ideal environment for a trial of an educational intervention in primary care, designed to improve clinical practice.

**Table 1:** Dementia indicators for the quality and outcomes framework

**Indicator**	**Description**
Dementia (DEM) indicator 1	The practice reports the number of patients with dementia on its register and the number of people with dementia as a proportion of its list size.
	Rationale: A register is a prerequisite for the organization of good primary care for a particular patient group. There is little evidence to support screening for dementia and it is expected that the diagnosis will largely be recorded from correspondence when patients are referred to secondary care with suspected dementia or as an additional diagnosis when a patient is seen in secondary care. However, it is also important to include patients where it is inappropriate or not possible to refer to a secondary care provider for a diagnosis and where the general practitioner has made a diagnosis based on their clinical judgment and knowledge of the patient.
Dementia (DEM) indicator 2	The percentage of patients diagnosed with dementia whose care has been reviewed by the practice in the preceding 15 months.
	Rationale: The face-to-face review should focus on support needs of the patient and their carer. In particular the review should address four key issues:
	(1) An appropriate physical and mental health review for the patient.
	(2) If applicable, the carer’s needs for information commensurate with the stage of the illness and his or her and the patient’s health and social care needs.
	(3) If applicable, the impact of caring on the caregiver.
	(4) Communication and co-ordination arrangements with secondary care (if applicable).
	A series of well-designed cohort and case control studies have demonstrated that people with Alzheimer-type dementia do not complain of common physical symptoms, but experience them to the same degree as the general population. Patient assessments should therefore include the assessment of any behavioral changes caused by: concurrent physical conditions (for example, joint pain or intercurrent infections) new appearance of features intrinsic to the disorder (for example, wandering) and delusions or hallucinations due to the dementia or as a result of caring behavior (for example, being dressed by a carer).
	Depression should also be considered since it is more common in people with dementia than those without the diagnosis and sources of help and support (bearing in mind issues of confidentiality).

Table adapted from the Quality and Outcomes Framework guidance 2009/10, BMA 2009 [14].

### Recognition of and response to dementia

This project builds on an earlier trial, which demonstrated that educational interventions can improve the recognition of dementia syndromes in general practice, but did not alter documented practice [15]. The educational intervention reported here was developed by a group of practitioners from relevant disciplines and advised by caregivers of people with dementia. The design process, which is described in detail elsewhere [16], was based on three premises:

1. An adult learning approach to solving real-world problems would be needed, emphasizing the educational benefits of conversations, understood as social acts of collaboration that generate and improvise ideas collectively [17].

2. Tailoring of learning to the needs of the learners can be achieved using processes of educational needs assessment [18] and educational prescription [19], both techniques developed by the evidence-based medicine movement [19]. Educational prescriptions are a useful tool for enabling follow-up of learning and can be used in any practice setting [20,21].

3. Learning would take place in the workplace, and be open to all team members (healthcare practitioners and administrative staff). Through reciprocal learning [22] peers and colleagues can be the most effective educators [22,23] and there is a particular benefit from learning from other disciplines [23,24]. This group effect in learning meant that a cluster-randomized design, with randomization at the practice level, was appropriate to this trial.

Adopting these premises, we developed a flexible learning needs assessment tool that allows an educational intervention to be tailored to the group needs and skills of the existing practice team. After a feasibility study [25] this intervention was tested in the current EVIDEM-ED randomized controlled trial [26].

## Methods

**Figure 1:**
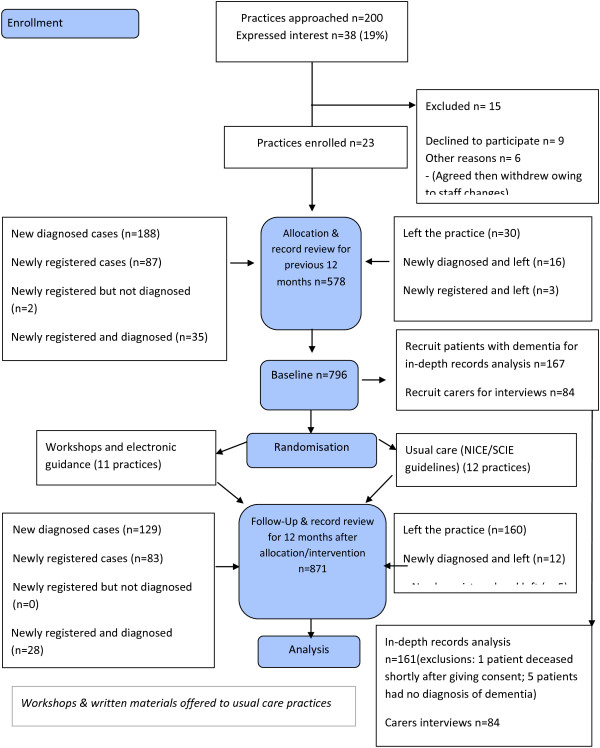
The EVIDEM-ED flow diagram.

The effectiveness of the new educational intervention was tested in an unblinded cluster randomized, controlled trial with a pre/post design and with two arms; usual care versus educational intervention (see consort diagram in Figure 1).

Standard significance tests assume that random sampling has taken place, and that the behavior or knowledge of any one individual is not affected by others in the sample. However, this study, like many health service evaluations, is based on clusters of individuals who may influence each other; in this case, colleagues in a practice who share information or similar views, or patients who receive similar treatment [27]. This 'clustering effect’ can mean that false conclusions are drawn about relationships in the data, an effect that we controlled for by using cluster membership (that is, respondents in each practice).

### Study setting

The study took place in group practices within the geographical care covered by the North Thames Dementias and Neurodegenerative Diseases Research Network (NT DeNDRoN).

### Educational intervention

The educational intervention consists of practice-based workshops designed to elicit and assess the educational needs for the team as a whole. Experienced general practitioners with a background in postgraduate education facilitated the small group workshops with the practice teams. This comprised of an initial needs assessment group discussion, which was guided by a set series of questions. The questions were designed to elicit information about what systems were already in place for the diagnosis of and care for dementia patients, but also to encourage all staff to reflect on what they were already doing, whether they were doing that well, what they still need to be doing, and how to fill the gaps in knowledge and service. The educational needs assessment questions were as follows:

1. How would you rate your current care for people with dementia and their carers (using a simple scale of good enough/satisfactory/needs substantial improvement)?

2. What grounds or criteria is your rating based on?

3. How do you arrive at your decision for diagnosis of dementia?

4. After diagnosis, what follow-up do you provide to people with dementia and their carers?

5. Are you using a shared care protocol for cholinesterase inhibitors? If 'yes’, then: (i) who was involved in producing the protocol; (ii) who is involved in its implementation (for example, hospital consultants, community psychiatric nurses (CPNs), Care of Older People team)?

6. How effective do you think cholinesterase inhibitors are and how effective have you found them in your practice?

7. What non-pharmacological alternatives do you have available to help your patients (and their carers)?

8. Based on your experience, what do you think are the important quality markers in caring for people with dementia? (What would you want for yourself?)

9. What would you like to improve in the care of patients with dementia, in your practice?

From this discussion, an educational prescription tailored to those needs was generated and supported with workshops and electronic information resources. The 'usual/normal’ care control practices were provided with a summary of the UK NICE/ Social Care Institute for Excellence (SCIE) dementia clinical guidelines [8] and were offered workshop training and electronic information resources at the end of the study. Practices were asked at the end of the study about any competing educational events.

### Recruitment

#### Primary care practices

Group practices in the North Thames DeNDRoN area were identified in collaboration with the local Primary Care Research Networks. Practices were contacted by the Trial research team, by letter and by awareness-raising through primary care educational meetings and by regular newsletters. Inclusion criteria for the practices were willingness to participate as a group in an educational experiment, and routine use of electronic medical records to capture the content of clinical encounters.

#### Patients

All practices were asked to identify patients with dementia by using electronic searches of their clinical record system updated by manual checks of the resulting list by medical and nursing staff.

#### Sample size

The size of the sample was based upon the following assumptions: (1) we expected a difference between the intervention and control groups in the proportion of patients with two or more dementia reviews in the 12 months after the educational intervention (or randomization to normal/usual care) of 50% (that is, control 20% versus 70% intervention); and (2) 20 practices would be included with an effective sample size of 10 patients per practice. Thus, a total effective sample size of 200 was reached.

This sample size was calculated based on randomization at the cluster (practice) level and allowed the study to maintain 90% power to detect the difference postulated in the presence of an intraclass correlation (ICC) of 0.37 and less.

### Outcome measurements

#### Primary outcome

Our hypothesis was that the proportion of patients receiving two dementia reviews per year (as recommended by guidelines as being clinically necessary [8]) would differ by 50%, (that is, 20% (usual care) versus 70% (intervention)) after the educational intervention. Electronic medical records were audited retrospectively for a period 12 months prior to intervention and again for the 12 months after intervention.

The primary outcome data was assessed by independent clinical research staff via an audit of the Dementia Indicators of the Quality Outcome Framework (see Table 1). Additional information collected was of the age, gender, date of diagnosis (pre or post trial randomization and/or intervention dates), diagnosis, and whether the person lived in their own home. (Individual patient consent was sought for a more detailed examination of the secondary outcome measures, reported elsewhere; see Figure 1). Clinicians independent of the practices classified management reviews for dementia in one of two ways; as planned, coded Dementia Annual Reviews eligible for inclusion in the Quality Outcomes Framework, and as opportunistic Dementia Reviews, which were documented reviews of any aspect of dementia during clinical encounters prompted by other problems. These clinical reviewers were trained in data extraction using a standardized data extraction form developed for the trial, and uncertainties about classification were discussed with the principal investigator.

### Randomization and masking

Participating practices were randomized to intervention or usual care arms by an independent researcher using a computer randomization program [28]. Independent clinicians undertaking record reviews could have deduced the allocation of the practices and could have become deblinded.

### Data collection

The trial lasted 36 months allowing for a follow-up period of 12 months to capture effects on clinical practice. The trial was conducted in accordance with Good Clinical Practice [29] (GCP) [26] and approval for the trial was received from Southampton & South West Hampshire Research Ethics Committee (A): reference 09/H0502/77.

Practices self-completed a data-recording form at randomization and at follow-up 12 months later. This comprised the following fields:

•Practice list size.

•Number of partners (full-time equivalent (FTE)).

•Number of practice nurses (FTE).

•Any other member of staff specific for caring for older people based within the practice? If so please state title.

•Do you currently look after residents in a nursing or care home? (if so how many patients have a dementia diagnosis? Do you include this number on your QOF reporting).

•Age/sex register:

Male 0 to 64 years

Female 0 to 64 years

Male 65+ years

Female 65+ years

•Practice deprivation score, (if known).

•Dem 1 QOF (no. of patients on register diagnosed with dementia).

•Dem 2 QOF (the percentage of patients diagnosed with dementia whose care has been reviewed in the previous 15 months).

The following anonymous audit data were collected by independent clinicians:

•Age at randomization and/or intervention.

•Registered with current practice pre or post randomization and/or intervention.

(If left) date left the practice

•Gender

•Living at home

•Diagnosis

•Date of diagnosis

•Number of dementia reviews within 12 months pre/post randomization and/or intervention

•Number of opportunistic dementia reviews within 12 months pre/post randomization and/or intervention

The data collection tools are available on request from the authors.

### Statistical analyses

We assessed the effect of the interventions at the practice level and analyses were performed on an intention to treat basis. We analyzed differences in detection rates by using binary logistic regression to include the cluster effect. These were calculated before and after the intervention, excluding cases previously diagnosed in another practice. Analysis of quantitative data was undertaken using the SPSS software package [30].

### Statistical modeling

Case identification: Multilevel Poisson regression modeling was used to compare the diagnosis rates between the intervention and control groups in the postintervention/randomization 12 months. The outcome used was the count of new diagnoses during this period (in those aged 65+) with the number of patients aged over 65 on the practice list used as the exposure variable. A two-level random coefficients model was fitted in order to take account of the cluster randomization. The model included a binary variable for group (0 = control, 1 = intervention). In order to take account of the differences in baseline rates the baseline counts in the 12 month preintervention/randomization period were included in the model.

Dementia management reviews: Multilevel logistic regression modeling was used to compare the proportion of patients who had two or more reviews in the postintervention or post-randomization 12 months, between the intervention and usual care groups. A separate model was fitted for each classification of reviews: planned; opportunistic; and total. Two-level random coefficient models were fitted in order to take account of the cluster randomization. The models included a binary variable for group (0 = control, 1 = intervention). In order to take account of the differences in baseline rates a cluster level variable was created indicating the proportion of patients within a cluster (practice) who had two or more reviews in the 12-month preintervention/randomization period.

Sensitivity Analysis: The above modeling was repeated with exclusion of those without the full 12 months follow-up in the postintervention/randomization period. Additionally, the cluster level variable indicating the proportion of patients within a practice with two or more reviews in the 12-month preintervention/randomization period was based only on those patients with the full 12 months of data (see Figure 1).

## Results

Of 200 practices approached, 23 agreed to participate and provide the required level of access and data. The practices came from 12 different primary care organizations in urban, semirural and rural areas. Practice enrolment occurred in 2008 to 2010.

As a result of the field-testing the variety of learning materials used were broadened to include more reference material given during sessions and available online for instant access. The pacing of delivery for topics was also amended and the expert tutors became more knowledgeable and aware of areas of need that were consistent across individuals and groups.

The intervention practice teams were offered flexible delivery of the needs assessment of workshop training and advised that 1-h sessions be the optimal length of each session. Practices had a mean of three educational workshops, including the needs assessment workshop at the beginning of the trial (range 2 to 4). These were staggered across the practices and took place from 2009 to 2011. The educational needs assessment generated individual workshop content for each practice. The workshops were delivered by one or two general practitioner dementia experts each working to a tutor’s manual that included learning objectives and timings. For three of the workshops a single trainer with non-participant observer facilitated the workshops (SI (3); FL (3)) and in four practices both facilitators were present (SI and FL). A non-participant observer (JW) took notes at all meetings to ensure that all educational needs were met and to check for consistency in delivery of the intervention.

Each practice defined their own team, which comprised the core clinical staff but with an extension of the intervention to administrative and support staff, community nursing teams and other professionals linked with the practice. We found this approach to work well with consistent attendance throughout the sessions from the teams. In several practices, the support staff attended the sessions and one practice invited community nursing staff and one representatives from the local paramedic team.

### Practice information

A total of 11 practices were randomized to the intervention arm and 12 to the usual care arm. The number of patients with dementia per practice ranged from 5 to 123 in the intervention arm and 6 to 108 in the usual care arm. The characteristics of the practices by arm of study are shown in Table 2. The differences between arms were: list size for the intervention arm; high deprivation score in the usual care arm group; number of patients resident in care homes in the intervention arm.

**Table 2:** Primary outcome analysis: practice characteristics by randomization group

**Variable**	**Summary measure**	**Intervention practices**	**Normal care practices**
Number of GPs	Mean (SD)	5.1 (2.1)	4.6 (2.7)
	Median	5	5.5
	Minimum	2	1
	Maximum	9	9
List size	Mean (SD)	8,382 (4,711)	7,892 (4,684)
	Median	6,849	9,239
	Minimum	2,682	1,133
	Maximum	19,323	14,358
Deprivation score^a^	Mean (SD)	20.4 (7.6)	19.9 (10.0)
	Median	22.0	17.5
	Minimum	8	7
	Maximum	29	40
Care homes	No. of practices with patients residing in care homes	9	9
	Minimum per practice	0	0
	Maximum per practice	15	6
	Total number homes group	30	17

^a^The Index of Multiple Deprivation (IMD) [31] is a standard measure of deprivation at small area level across England. The IMD is based on seven domains: income, employment, health and disability, education and skills, barriers to housing and services, living environment, and crime. The scores used in the indices are relative to each other and (in most cases) do not indicate an absolute value as such. For example, an IMD score of 40 does not mean that an area is twice as deprived as an area with a score of 20, but it does mean that the area with the score of 40 is more deprived than the area with a score of 20.

### Patient information

A total of 1,072 (intervention: 512, control: 560) patients had information available in their medical records showing the number of reviews (planned/opportunistic/total) within 12 months (or a proportion of) before intervention or randomization and/or within the 12 months (or a proportion of) after. The majority were female: intervention group 61% (N = 313), control group 70% (N = 382). The mean age for those people with dementia in the intervention group was 83 years (SD 8.7; minimum 33; maximum 104) and 83 years (SD 8.8; minimum 55; maximum 109) for those in the control group.

### Dementia management reviews

The mean total number of dementia management reviews (planned and opportunistic) for people with dementia in the intervention group in the period before intervention was 0.89 (SD 0.92; minimum 0; median 1; maximum 4). For the period after intervention it was 0.89 (SD 1.09; minimum 0; median 1; maximum 8).

For those people with dementia in the control group prior to randomization the mean total number of dementia management reviews (planned and opportunistic) was 1.66 (SD 1.87; minimum 0; median 1; maximum 12). For the period after randomization it was 1.56 (SD 1.79; minimum 0; median 1; maximum 11).

### Primary outcomes

The numbers of each type of review for each patient in the pre/post periods were dichotomized for the primary analyses. These were classified according to whether the individual had <2 or 2 ≥ dementia management reviews; summarized in Table 3 below.

**Table 3:** Percentage of patients, by group, with 2 ≥ reviews for each type of review in the pre/post periods

**Variable**	**Intervention practice patients**	**Control practice patients**
Dementia (preintervention)	4.9%	15.1%
Dementia (postintervention)	6.1%	9.6%
Opportunistic (preintervention)	6.6%	21.3%
Opportunistic (postintervention)	8.3%	21.4%
Total (preintervention)	18.2%	39.0%
Total (postintervention)	19.8%	35.9%

Estimated odds ratios (odds of having two or more reviews in the intervention versus the usual care group), along with the *P* value and 95% confidence intervals for those with a full and partial data period are presented in Table 4.

**Table 4:** **Estimated odds, *P *** **value and 95% confidence intervals by classification of review**

**Reviews (≥2 versus <2)**	**Odds ratio**	**95% confidence interval**	***P *** **value**
For all cases including proportion of data collection period pre/post-intervention			
Dementia	0.94	0.33 to 2.62	0.899
Opportunistic	0.96	0.53 to 1.74	0.890
Total	1.05	0.72 to 1.53	0.811
For full pre/post-intervention data period			
Dementia	0.83	0.32 to 2.10	0.688
Opportunistic	0.62	0.25 to 1.56	0.310
Total	0.83	0.52 to 1.33	0.444

There was no significant difference in recording of dementia management reviews for patients diagnosed by the current practice before or after intervention.

### Case detection

Preintervention period: In the preintervention/randomization 12 months period there were a total of 239 newly diagnosed cases of dementia. Of these, 11 (4.6%) were in people aged below 65. Of the 228 newly diagnosed cases in those aged 65 and over 99 were in the control practices and 129 in the intervention practices.

Postintervention period: In the postintervention/randomization 12 months period there were a total of 169 newly diagnosed cases of dementia. Six of these (3.7%) were in people aged below 65. Of the 163 newly diagnosed cases in those aged 65 and over, 78 were in the control practices and 85 in the intervention practices.

The primary analysis included diagnosis rates in those aged 65 and over. Each practice provided data for those aged 65 and over registered with the practice list and this was used as the denominator for the calculation of rates. For the control practices combined the total patient population was 15,699 versus 11,541 for the intervention practices.

Table 5 shows case detection rates in the pre/post-intervention/randomization periods. These are shown separately for the combined intervention practices and the combined usual care practices; also the minimum and maximum rates were calculated across the practices.

**Table 5:** Detection rates for new cases of dementia pre/post-intervention period by randomization arm

**Period**	**Rates**	**Intervention practices**	**Control practices**
Preintervention period	Combined	1.12%	0.63%
	Minimum	0.17%	0%
	Maximum	3.45%	4.4%
Postintervention period	Combined	0.74%	0.50%
	Minimum	0%	0%
	Maximum	1.06%	4.1%

Case detection rates were unaffected by the intervention. The estimated incidence rate ratio (IRR) for the intervention versus the control group from the multilevel Poisson regression modeling was 1.03; the *P* value was 0.927 with 95% confidence intervals 0.57, 1.86.

Case detection rates were unaffected by the intervention. The estimated incidence rate ratio (IRR) for the intervention versus the control group from this model was 1.03; the *P* value was 0.927 with 95% confidence intervals 0.57, 1.86.

## Discussion

The English policy imperatives and financial incentives for dementia diagnosis and management have created a favorable environment for a trial of an educational intervention designed to improve clinical practice in primary care. The educational intervention was developed following the Medical Research Council’s recommendations for complex interventions [32], with strong elements of codesign modified by nominal groups to gain the insights and experiences of a range of practitioners [33]. Codesign is a technique adopted from product development, which has tangible benefits in developing or redesigning health services [34-37]. The educational needs assessment deployed in this trial is an example of a strategy aimed to improve quality of care by overcoming the translation block that obstructs the diffusion of clinical guidelines and knowledge into practice [38]. In this study, we found no significant improvement in case identification or documentation of dementia management reviews after an educational intervention tailored to practice educational needs, despite the financial incentives to identify and follow-up patients with dementia. There are several possible reasons for this.

The intervention may have been too weak to change practice. More workshops may have been needed, with reinforcement or mentoring of practitioners over longer periods of time. This level of educational input was not practicable in this trial, and we doubt that it would be feasible in real-world primary care organizations. Physicians have a limited ability to accurately self-assess their competence [39]. Although the educational needs assessment was designed as a group process to offset this tendency, more external assessment may have been needed to truly tailor the intervention to needs.

It is possible that the trial was underpowered for the 50% expected change. Other changes may be detectable. Professional knowledge, confidence and attitudes; Dementia management activity concordant with the NICE guidelines and carers’ satisfaction and unmet need were all measured pre and post intervention and will be reported elsewhere. It is possible that these or other unmeasured outcomes (such as patient satisfaction with care) may have had an impact as a result of the intervention.

### Limitations of the study

It is possible that using medical record coded QOF management reviews as the primary outcome did not capture changes in dementia management. However, our creation of a category of 'opportunistic dementia review’ fitted with clinical practice and allowed a generous interpretation of clinical activity. Additionally, many patients with dementia joined or left during the pre/post periods, truncating the data collection time, so that length of follow-up may have been too short to capture a difference.

The study took place in the South East of England, with practices that were probably innovative early adopters, not typical general practices, and local educational programs developed to implement the National Dementia Strategy may have influenced practice activities, although we found no evidence of this. The volunteer practices were probably different from others, in that they wanted to take part in a pilot educational program about dementia. However, the results of this study have wider implications, particularly about the value of tailoring educational interventions. Distribution of newsletters and guidelines to normal care arm practices may have had an effect on their behavior.

The intervention was developed in ways consistent with current understanding of how effective interventions are made. Nevertheless, there may have been deficiencies in the development process. For example, the views of people with dementia and their family carers may not have had sufficient weight. Some professional perspectives may have been too powerful, resulting in an oversimplification of the educational needs assessment.

## Conclusions

This study suggests that a tailored educational intervention aimed at general practitioners does not improve documentation of clinical management for people with dementia, or dementia case identification, even when policy pressure and consumer demand encourage changes in clinical practice and the reimbursement system rewards it. Educational interventions in settings where a coordinated system of dementia case management operated have shown positive effects [32], so the effective change may need to include the additional resource of case management as well as focused education.

## Competing interests

The authors declare that they have no competing interests.

## Authors’ contributions

SI, JW and MG conceived the study and obtained funding for it. JW was trial manager, PJ was trial administrator, SI, JW, FL, PJ and IT-B took part in practice workshops, DR extracted data and MG was the trial statistician; all contributed to the drafting and final approval of this paper.

## References

[B1] Bamford C Lamont S Eccles M Robinson L May C Bond J Disclosing a diagnosis of dementia: a systematic review Int J Geriatr Psychiatry 2004 19 151 169 10.1002/gps.1050 14758581

[B2] Pratt R Wilkinson H A psychosocial model of understanding the experience of receiving a diagnosis of dementia Dementia 2003 2 181 199 10.1177/1471301203002002004

[B3] Husband HJ The psychological consequences of learning a diagnosis of dementia: three case examples Aging Ment Health 1999 3 179 183 10.1080/13607869956352

[B4] Smith AP Beattie BL Disclosing a diagnosis of Alzheimer’s disease: patients and families’ experiences Can J Neurol Sci 2001 28 Suppl 1 67 71 10.1017/s0317167100001220 11237313

[B5] Pucci E Angeleri F Borsetti G Brizioli E Cartechini E Giuliani G Solari A General practitioners facing dementia: are they fully prepared? Neurol Sci 2004 24 384 389 10.1007/s10072-003-0193-0 14767683

[B6] Vernooij-Dassen M Moniz-Cook E Woods R De Lepeleire J Leuschner A Zanetti O de Rotrou J Kenny J Franco M Peters V Iliffe S INTERDEM group: Factors affecting the timely recognition and diagnosis of dementia in primary care across eight European states: a modified focus group study Int J Geriatr Psychiatry 2005 20 1 10 10.1002/gps.1255 15799080

[B7] Boustani M Peterson B Hanson L Harris R Lohr KN U.S. Preventive Services Task Force Screening for dementia in primary care: a summary of the evidence for the US preventive services task force Ann Int med 2003 138 927 937 12779304 10.7326/0003-4819-138-11-200306030-00015

[B8] NICE NICE clinical guideline 42: Dementia. Supporting people with dementia and their carers in health and social care [http://guidance.nice.org.uk/CG42/NICEGuidance/pdf/English]

[B9] Turner S Iliffe S Downs M Wilcock J Bryans M Levin E Keady J O'Carroll R General practitioners’ knowledge, confidence and attitudes in the diagnosis and management of dementia Age Ageing 2004 33 461 467 10.1093/ageing/afh140 15271637

[B10] Iliffe S Wilcock J The identification of barriers to the recognition of and response to dementia in primary care using a modified focus group method Dementia 2005 4 73 85 10.1177/1471301205049191

[B11] Perry M Drascovic I Lucassen P Vernooij-Dassen M van Achterberg T Rikkert M Effects of educational interventions on primary dementia care: systematic review Int J Geriatr Psychiatry 2011 26 1 11 10.1002/gps.2479 21157845

[B12] Department of Health National Dementia Strategy 2009 London, UK: DoH

[B13] BMA Quality and Outcomes Framework guidance for GMS contract 2011/12: Delivering investment in general practice [http://www.nhsemployers.org/Aboutus/Publications/Documents/QOF_guidance_GMS_contract_2011_12.pdf]

[B14] BMA Quality and Outcomes Framework guidance for GMS contract 2009/10 [http://www.hscic.gov.uk/qof]

[B15] Downs M Turner S Iliffe S Bryans M Wilcock J Keady J Levin E O’Carroll RE Howie K Iliffe S Effectiveness of educational interventions in improving detection and management of dementia in primary care: cluster randomized controlled study BMJ 2006 332 692 696 10.1136/bmj.332.7543.692 16565124 PMC1410839

[B16] Iliffe S Jain P Wong G Lefford F Gupta S Warner A Kennedy H Dementia diagnosis in primary care: thinking outside the educational box Aging Health 2009 5 51 59 10.2217/1745509X.5.1.51

[B17] Jordan ME Lanham HJ Crabtree BF Nutting PA Miller WL Stange KC McDaniel RR The role of conversation in health care interventions: enabling sensemaking and learning Implementation Science 2009 4 15 10.1186/1748-5908-4-15 PMC2663543 19284660

[B18] Grant J Learning needs assessment: assessing the need BMJ 2002 324 156 159 10.1136/bmj.324.7330.156 11799035 PMC64520

[B19] Green M Evaluating evidence based practice performance Evid Based Med 2006 11 99 100 10.1136/ebm.11.4.99 17213114

[B20] Sackett D Straus S Finding and applying evidence during clinical rounds JAMA 1998 280 1336 1338 10.1001/jama.280.15.1336 9794314

[B21] Khunti K Teaching evidence-based medicine using educational prescriptions in general practice Med Teach 1998 20 380 381 10.1080/01421599880841

[B22] Leykum LK Palmer R Lanham H Jordan M McDaniel RR Noel PH Parchman M Reciprocal learning and chronic care model implementation in primary care BMC Health Serv Res 2011 11 44 10.1186/1472-6963-11-44 21345225 PMC3050698

[B23] Nowlem PM A New Approach to Continuing Education for Business and the Professions 1988 New York, NY: MacMillan

[B24] Grant J Stanton F The effectiveness of continuing professional development 2000 Edinburgh, UK: The Association for the Study of Medical Education

[B25] Iliffe S Koch T Jain P Lefford F Wong G Warner A Wilcock J Developing an educational intervention on dementia diagnosis and management in primary care for the EVIDEM-ED trial Trials 2012 13 142 10.1186/1745-6215-13-142 22913431 PMC3492020

[B26] Iliffe S Wilcock J Griffin M Jain P Thuné-Boyle I Koch T Lefford F Evidence-based interventions in dementia: a pragmatic cluster-randomised trial of an educational intervention to promote earlier recognition and response to dementia in primary care (EVIDEM-ED) Trials 2010 11 13 10.1186/1745-6215-11-13 20146803 PMC2829558

[B27] Ukoumunne OC Gulliford MC Chinn S Sterne JAC Burney PCJ Methods for evaluating area-wide and organisation-based interventions in health and health care: a systematic review Health Technol Assess 1999 3 5 10982317

[B28] Research Randomizer Research Randomizer, a free online tool available for researchers [http://www.randomizer.org]

[B29] Medical Research Council Good Clinical Practice (GCP) in Clinical Trials 1998 London, UK: MRC

[B30] IBM SPSS Statistics [http://www-01.ibm.com/software/uk/analytics/spss/]

[B31] British Department for Communities and Local Government The indices of deprivation 2010 (ID 2010) (DCLG) [https://www.gov.uk/government/uploads/system/uploads/attachment_data/file/6871/1871208.pdf]

[B32] Medical Research Council A Framework for Development and Evaluation of RCTs for Complex Interventions to Improve Health 2000 London, UK: MRC

[B33] Kaulio M Customer, consumer and user involvement in product development: a framework and a review of selected methods Total Qual Manag Bus Excell 1998 9 141 149

[B34] Paton N Callander R Cavill M Ning L Weavell W Collaborative quality improvement: consumers, carers and mental health service providers working together in service co-design Australas Psychiatry 2013 21 78 79 10.1177/1039856212465347 23344806

[B35] Piper D Ledema R Gray J Verma R Holmes L Manning N Utilizing experience-based co-design to improve the experience of patients accessing emergency departments in New South Wales public hospitals: an evaluation study Health Serv Manage Res 2012 25 162 172 10.1177/0951484812474247 23554443

[B36] Boyd H McKernon S Mullin B Old A Improving healthcare through the use of co-design N Z Med J 2012 125 76 87 22854362

[B37] Bate P Robert G Experience-based design: from redesigning the system around the patient to co-designing services with the patient Qual Saf Health Care 2006 15 307 310 10.1136/qshc.2005.016527 17074863 PMC2565809

[B38] Rubinstein L Pugh J Strategies for promoting organizational practice change by advancing implementation research J Gen Intern Med 2006 21 S58 S64 10.1111/j.1525-1497.2006.00364.x PMC2557137 16637962

[B39] Davis D Mazmanian P Fordis M van Harrison R Thorpe K Perrier L Accuracy of physician self-assessment compared with observed measures of competence JAMA 2006 296 1094 1102 10.1001/jama.296.9.1094 16954489

